# Postoperative clinical outcomes of patients with thymic epithelial tumors after over-3-year follow-up at a single-center

**DOI:** 10.1186/s13019-023-02169-6

**Published:** 2023-02-21

**Authors:** Peng Jiao, Wen-Xin Tian, Fan-Juan Wu, Yu-Xing Liu, Jiang-Yu Wu, Yao-Guang Sun, Han-Bo Yu, Chuan Huang, Qing-Jun Wu, Chao Ma, Dong-Hang Li, Hong-Feng Tong, Jun Li

**Affiliations:** 1 Department of Thoracic Surgery, Beijing Hospital, National Center of Gerontology; Institute of Geriatric Medicine, Chinese Academy of Medical Sciences, Beijing, People’s Republic of China; 2 Peking University Health Science Center, Beijing, People’s Republic of China; 3 Department of Thoracic Surgery, Provincial Hospital Affiliated to Shandong First Medical University, No. 324, Jing 5 Wei 7 Road, Jinan, 250021 Shandong People’s Republic of China

**Keywords:** Thymic epithelial tumor, Thymectomy, Myasthenia gravis, Prognosis

## Abstract

**Background:**

To evaluate postoperative clinical outcomes and analyze influencing factors for patients with thymic epithelial tumors over 3 years after operation.

**Methods:**

Patients with thymic epithelial tumors (TETs) who underwent surgical treatment in the Department of Thoracic Surgery at Beijing Hospital from January 2011 to May 2019 were retrospectively enrolled in the study. Basic patient information, clinical, pathological, and perioperative data were collected. Patients were followed up by telephone interviews and outpatient records. Statistical analyses were performed using SPSS version 26.0.

**Results:**

A total of 242 patients (129 men, 113 women) with TETs were included in this study, of which 150 patients (62.0%) were combined with myasthenia gravis (MG) and 92 patients (38.0%) were not. 216 patients were successfully followed up and their complete information was available. The median follow-up period was 70.5 months (range, 2–137 months). The 3-year overall survival (OS) rate of the whole group was 93.9%, and the 5-year OS rate was 91.1%. The 3-year relapse-free survival (RFS) rate of the whole group was 92.2%, and the 5-year relapse-free survival rate was 89.8%. Multivariable COX regression analysis indicated that recurrence of thymoma was an independent risk factor for OS. Younger age, Masaoka-Koga stage III + IV, and TNM stage III + IV were independent risk factors for RFS. Multivariable COX regression analysis indicated that Masaoka-Koga staging III + IV, WHO type B + C were independent risk factors for postoperative improvement of MG. For patients with MG, the postoperative complete stable remission (CSR) rate was 30.5%. And the result of multivariable COX regression analysis showed that thymoma patients with MG with Osserman staging IIA + IIB + III + IV were not prone to achieving CSR. Compared with patients without MG, MG was more likely to develop in patients with WHO classification type B, and patients with myasthenia gravis were younger, with longer operative duration, and more likely to develop perioperative complications.

**Conclusions:**

The 5-year overall survival rate of patients with TETs was 91.1% in this study. Younger age and advanced stage were independent risk factors for RFS of patients with TETs, and recurrence of thymoma were independent risk factors for OS. In patients with MG, WHO classification type B and advanced stage were independent predictors of poor outcomes of MG treatment after thymectomy.

**Supplementary Information:**

The online version contains supplementary material available at 10.1186/s13019-023-02169-6.

## Background

Thymic epithelial tumors (TET) originate from the thymic epithelium and are low-malignant tumors with different pathological types and biological behaviors, including thymoma and thymic carcinoma. TET is one of the most common tumors in the anterior mediastinum, accounting for approximately 15% of mediastinal tumors in adults [1]. Thymomas are often associated with various autoimmune diseases, of which myasthenia gravis is the most prevalent. Surgical resection has been the mainstay of therapy for thymic epithelial tumors [2–4]. The traditional surgical procedure is median sternotomy, while thoracoscopic thymectomy has been accepted in recent years for its minimal invasiness, safety, and effectiveness [5, 6]. The incidence of TET is quite low, and there are few single-center reports of long-term follow-ups with large sample sizes after surgical resection.

## Methods

### Patients

Our study was approved by the Institutional Ethics Review Board of Beijing Hospital. Informed consent was waived due to the retrospective nature of the study.

In this study, patients with thymic epithelial tumors who underwent surgical treatment in the Department of Thoracic Surgery at Beijing Hospital from January 2011 to May 2019 were selected. A total of 242 patients (129 men and 113 women) were included. 216 patients were successfully followed up, all of whom were followed up for more than 3 years, and their complete information was available. The median follow-up time was 70.5 months (range: 2–137 months, individually less than 3 years because several patients died during the follow-up period). 26 cases (10.74%) were lost to follow-up. 150 patients had tumors combined with myasthenia gravis and 92 patients did not have tumors combined with myasthenia gravis.

### Surgical techniques

Two kinds of surgical procedures were performed in this study, including conventional median sternotomy for thymus and thymoma resection and thoracoscopic thymectomy. When the diameter of the tumor was larger than 5 cm or invaded important blood vessels (the innominate vein, superior vena cava, aorta), median sternotomy was usually chosen. Otherwise, thoracoscopic surgery was the first choice, including thoracoscopy via the right side of the chest, thoracoscopy via the left side of the chest, and subxiphoid and subcostal thoracoscopy. Extended thymectomy was performed for patients with MG, which was defined as the removal of the tumor and thymus with anterior mediastinal fat tissue bilaterally between the phrenic nerves, from the root of the neck to the diaphragm. Patients without MG received thymectomy, which was defined as the removal of the tumor and the entire thymus. Ivaded tissues such as lung tissue, pericardium, and phrenic nerve may also be resected for complete resection of the tumor. Surgery was defined as radical (R0) when a complete tumor resection was performed, and incomplete in the case of microscopically (R1) or macroscopically (R2) residuals.

### Postoperative treatment

Radiotherapy was recommended for patients with tumor invasion to the capsule or more advanced stages according to postoperative pathology. For patients with Masaoka stage IV or R2 resection, postoperative radiotherapy and chemotherapy were recommended.

### Observation index and data collection

Information was observed carefully and recorded detailedly, including age, preoperative course, Charlson Comorbidity Index (CCI), tumor size, presence of MG and myasthenia gravis symptom staging (Osserman classification system), surgical approach, operative time, intraoperative bleeding, postoperative drainage, pathological findings, WHO classification, Masaoka-Koga stage, whether complete resection or not, postoperative complications, and other clinical indicators. Patients were followed up every 6 months in the first year after surgery and thereafter once a year by telephone interviews and outpatient records. Clinical indicators such as postoperative treatment, recurrence, survival status, and MG improvement were collected.

### Statistical analysis

Statistical analyses were performed using SPSS version 26.0 statistical software. For continuous variables, the normality test needs to be performed. Normally distributed continuous data is presented as mean ± standard deviation using the t-test and not normally distributed using the Mann–Whitney U test are described as categorical variables recorded in the form of median (quartiles) [M (P25, P75)], and χ2 test or Fisher exact tests are conducted. Risk factors for postoperative survival of patients were analyzed using Cox proportional risk models. Risk factors of thymoma recurrence after surgery were analyzed using univariate and multifactorial Cox regression analysis. All statistical tests were bivariate and *P* values < 0.05 were considered statistically significant.

## Results

### Basic clinical data

A total of 242 patients (129 men, 53.3%; 113 women, 46.7%) with thymic epithelial tumors were included. The mean age of the patients was 52.7 ± 13.7 years. 150 patients (62.0%) were combined with myasthenia gravis, and 92 patients (38.0%) were not. We assessed each patients using CCI, which resulted as the following: 168 Score 0(69.4%), 57 Score 1(23.6%), 16 Score 2(6.6%) and 1 Score 3(0.4%). The mean diameter of tumors was 4.4 ± 2.2 cm.149 patients received thoracoscopic surgery while 93 patients received median sternotomy. 230 cases received R0 resection, 6 cases R1 resection, and 6 cases R2 resection. And the average operation duration was 125.0 ± 48.1 min. According to WHO pathological classification, there were 10 type A (4.1%), 45 type AB (18.6%), 47 type B1 (19.4%), 75 type B2 (31.0%), 13 type B3 (5.4%), 32 mixed B types (13.2%), 4 type MNT (2.0%), and 1 unknown (0.5%). According to Masaoka-Koga staging, there were 50 stage I (20.7%), 41 stage IIA (16.9%), 82 stage IIB (33.9%), 52 stage III (21.5%), and 17 stage IV (7.0%).

Within 1 month after surgery, one patient died of pneumonia caused by myasthenia gravis crisis, and there were no other perioperative deaths. A total of 34 patients developed postoperative complications. 18 patients with MG suffered from postoperative myasthenic crisis. 16 patients developed other complications, including 2 incisional infections, 2 bleeding requiring secondary surgery, 1 postoperative blood transfusion because of drainage volume > **ml, 4 atrial fibrillations, 1 heart infarction, 1 cardiac insufficiency, 3 deep vein thrombosis, 1 chylothorax, and 1 lower limb artery embolization.

A total of 134 patients received postoperative adjuvant therapy, of whom 114 underwent radiotherapy, 6 underwent chemotherapy, 6 underwent radiotherapy and chemotherapy, and 8 underwent other therapy.

### Follow-up

Of the 242 patients, 216 were successfully followed up. The median follow-up period was 70.5 months (range: 2–137 months). By the end of follow-up, a total of 23 patients died, of which 9 (42.9%) were directly or indirectly caused by MG exacerbation, 8 (38.1%) by recurrence of thymic epithelial tumors, 1 by heart infarction, 1 by other malignancies, 1 by pneumonia, and 1 unknown. A total of 28 patients relapsed. The 3-year overall survival (OS) rate of the whole group of patients was 93.9%, and the 5-year OS rate was 91.1%. The 3-year relapse-free survival (RFS) rate of the whole group of patients was 92.2%, and the 5-year RFS rate was 89.8%. The K-M survival curves of OS and RFS for the whole group of patients are shown in Figure S1: The K-M survival curves of OS and RFS for patients (see in Additional file 1). Basic clinical data is shown in Table 1.

**Table 1:** Basic clinical data

Variables	Patients without MG
N	242
Sex (male/female)	129/113
Age (year)	52.7 ± 13.7
Tumor size (cm)	4.4 ± 2.2
Operating duration (min)	125.0 ± 48.1
Intraoperative blood loss (ml)	50 (10–2000)
Surgical approach (OT/VATS)	93/149
WHO pathological classification (n (%))	
A	10 (4.1%)
AB	45 (18.6%)
MNT	4 (1.7%)
B1	47 (19.4%)
B2	75 (31.0%)
B3	13 (5.4%)
C	15 (6.2%)
MIX	32 (13.2%)
Unknown	1 (0.4%)
Masaoka-Koga staging (n (%))	
I	50 (20.7%)
IIA	41 (16.9%)
IIB	82 (33.9%)
III	52 (21.5%)
IV	17 (7.0%)
Resection status (R0/R1/R2)	230/6/6
Perioperative complications (n (%))	36 (14.9%)
Postoperative therapy (n (%))	134 (55.4%)
Types of postoperative therapy (n (%))	
Radiotherapy	114 (47.1%)
Chemotherapy	6 (2.5%)
Chemoradiotherapy	6 (2.5%)
Others	7 (2.9%)

#### Risk factors for thymoma survival

Sex, age, presence of MG, WHO pathological classification, Masaoka-Koga staging, surgical approach, resection status, presence of perioperative complications, and whether recurrence or not were included as variables in the COX regression analysis. Univariate COX regression analysis indicated that Charlson Comorbidity Index (*P* = 0.016), Masaoka-Koga staging (*P* = 0.000), TNM staging (*P* = 0.000), non-R0 resection (*P* = 0.000), perioperative complications (*P* = 0.017), postoperative therapy (*P* = 0.067) and recurrence of thymoma (*P* = 0.000) were risk factors for survival. Variables that showed a *P* value < 0.1 in univariate Cox regression analysis were entered into a multivariate Cox proportional-hazards regression model. The result of multivariable COX regression analysis proved that recurrence of thymoma was an independent risk factor for survival (*P* = 0.002/0.005). (Table 2).

**Table 2:** Univariate and multivariate Cox regression analysis of risk factors for thymoma survival

Variables	Univariate model			Multivariate model		
	OR	95% CI	*P* value	OR	95% CI	*P* value
Sex						
Female	Reference					
Male	1.329	0.568–3.111	0.512			
Age	1.013	0.982–1.045	0.418			
Myasthenia gravis	1.024	0.429–2.445	0.958			
CCI						
0	Reference			Reference		
1&2&3	2.793	1.206–6.468	0.016	2.169	0.827–5.697	0.116
WHO pathological classification						
A + AB + MNT	Reference					
B + MIX + C	2.249	0.665–7.602	0.192			
Masaoka-Koga staging						
I + II	Reference			Reference		
III + IV	8.472	3.307–21.704	0.000	2.011	0.420–9.637	0.382
Surgical approach						
OT	Reference					
VATS	0.492	0.209–1.160	0.105			
Resection status						
R0	Reference			Reference		
None R0	6.818	2.511–18.518	0.000	0.736	0.188–2.880	0.660
Perioperative complications	3.070	1.224–7.700	0.017	1.597	0.563–4.531	0.379
Postoperative therapy	2.766	0.930–8.222	0.067	1.160	0.277–4.855	0.839
Recurrence	16.011	6.297–40.711	0.000	7.826	1.842–33.250	0.005

Variables	Univariate model			Multivariate model		
	OR	95% CI	*P* value	OR	95% CI	*P* value
Sex						
Female	Reference					
Male	1.329	0.568–3.111	0.512			
Age	1.013	0.982–1.045	0.418			
Myasthenia gravis	1.024	0.429–2.445	0.958			
CCI						
0	Reference			Reference		
1&2&3	2.793	1.206–6.468	0.016	2.129	0.805–5.634	0.128
WHO pathological classification						
A + AB + MNT	Reference					
B + MIX + C	2.249	0.665–7.602	0.192			
TNM staging						
I + II	Reference			Reference		
III + IV	7.622	3.106–18.704	0.000	1.513	0.337–6.792	0.589
Surgical approach						
OT	Reference					
VATS	0.492	0.209–1.160	0.105			
Resection status						
R0	Reference			Reference		
None R0	6.818	2.511–18.518	0.000	0.723	0.183–2.851	0.643
Perioperative complications	3.070	1.224–7.700	0.017	1.590	0.551–4.592	0.391
Postoperative therapy	2.766	0.930–8.222	0.067	1.262	0.310–5.136	0.745
Recurrence	16.011	6.297–40.711	0.000	9.433	2.235–39.818	0.002

#### Risk factors for recurrence

Sex, age, presence of MG, WHO pathological classification, Masaoka-Koga staging, surgical approach, resection status, presence of perioperative complications, and perioperative complications were included as variables in the COX regression analysis. Univariate COX regression analysis indicated that younger age (*P* = 0.002), WHO pathological classification B + MIX + C (*P* = 0.081), Masaoka-Koga staging III + IV (*P* = 0.000), TNM staging III + IV (*P* = 0.000), non-R0 resection (*P* = 0.000), perioperative complications (*P* = 0.011) and postoperative therapy (*P* = 0.009) were risk factors for recurrence. Variables that showed a *P* value < 0.1 in univariate Cox regression analysis were entered into a multivariate Cox proportional-hazards regression model. The result of multivariable COX regression analysis proved that younger age (*P* = 0.002), Masaoka-Koga staging III + IV (*P* = 0.000) and TNM staging III + IV (*P* = 0.000) were independent risk factors for recurrence. (Table 3).

**Table 3:** Univariate and multivariate cox regression analysis of recurrence-free survival in patients with thymoma

Variables	Univariate model			Multivariate model		
	OR	95% CI	*P* value	OR	95% CI	*P* value
Sex						
Female	Reference					
Male	1.833	0.777–4.324	0.166			
Age	0.953	0.925–0.982	0.002	0.941	0.904–0.979	0.002
Myasthenia gravis	2.127	0.789–5.738	0.136			
CCI						
0	Reference					
1&2&3	1.543	0.667–3.568	0.311			
WHO pathological classification						
A + AB + MNT	Reference			Reference		
B + MIX + C	32.769	0.652–1647.895	0.081	-	-	0.959
Masaoka-Koga staging						
I + II	Reference			Reference		
III + IV	39.471	9.235–168.708	0.000	46.241	5.927–360.755	0.000
Surgical approach						
OT	Reference					
VATS	0.604	0.264–1.381	0.232			
Resection status						
R0	Reference			Reference		
None R0	13.604	5.255–35.216	0.000	1.567	0.551–4.454	0.400
Perioperative complications	3.265	1.316–8.103	0.011	1.785	0.667–4.778	0.249
Postoperative therapy	14.282	1.925–105.974	0.009	4.369	0.481–39.665	0.190

Variables	Univariate model			Multivariate model		
	OR	95% CI	*P* value	OR	95% CI	*P* value
Sex						
Female	Reference					
Male	1.833	0.777–4.324	0.166			
Age	0.953	0.925–0.982	0.002	0.941	0.904–0.979	0.002
Myasthenia gravis	2.127	0.789–5.738	0.136			
CCI						
0	Reference					
1&2&3	1.543	0.667–3.568	0.311			
WHO pathological classification						
A + AB + MNT	Reference			Reference		
B + MIX + C	32.769	0.652–1647.895	0.081	-	-	0.961
TNM staging						
I + II	Reference			Reference		
III + IV	20.646	7.008–60.822	0.000	16.814	4.529–62.421	0.000
Surgical approach						
OT	Reference					
VATS	0.604	0.264–1.381	0.232			
Resection status						
R0	Reference			Reference		
None R0	13.604	5.255–35.216	0.000	1.516	0.527–4.365	0.440
Perioperative complications	3.265	1.316–8.103	0.011	1.588	0.600–4.199	0.352
Postoperative therapy	14.282	1.925–105.974	0.009	5.536	0.649–47.222	0.118

#### Analysis of the factors influencing the treatment effect of MG

We have successfully followed up 150 thymoma patients with MG. Finally, 131 patients were included to evaluate the post-intervention status of MG. A total of 40 patients (30.5%) achieved complete stable remission (CSR), 5 patients (3.8%) achieved pharmacologic remission (PR), 38 patients (29.0%) had minimal manifestations (MM), 26 patients (19.8%) had improved (I), 6 patients (4.6%) were unchanged (U), 4 patients (3.1%) were worse (W), four patients (3.1%) had exacerbation (E), and 8 patients (6.1%) died of MG (D). The overall effective rate, including CSR, PR, MM, and I, was 78.6% (103/131).

The Cox regression analysis of postoperative improvement (the proportion of CSR + PR + MM + I) is shown below. Univariate COX regression analysis indicated that Masaoka-Koga staging III + IV (*P* = 0.010), TNM staging III + IV (*P* = 0.045) and WHO pathological classification B + C (*P* = 0.019) were risk factors for postoperative improvement. The result of multivariable COX regression analysis proved that Masaoka-Koga staging III + IV (*P* = 0.023) and WHO pathological classification B + C (*P* = 0.038/0.056) were independent risk factors for postoperative improvement. (Table 4).

**Table 4:** Analysis of factors influencing postoperative MG treatment in thymoma patients with MG

Variables	Univariate model			Multivariate model		
	OR	95% CI	*P* value	OR	95% CI	*P* value
Sex						
Female	Reference					
Male	0.972	0.666–1.420	0.884			
Age	1.006	0.993–1.020	0.357			
Preoperative course						
< 12 month	Reference					
≥ 12 month	0.823	0.498–1.359	0.447			
Application of hormone	0.821	0.414–1.628	0.572			
Application of immunosuppressants	1.001	0.501–1.997	0.998			
Osserman classification						
I + IIA	Reference					
IIB + III + IV	0.836	0.569–1.2	0.361			
WHO pathological classification						
A + AB + MNT	Reference			Reference		
B + MIX	0.508	0.321–0.904	0.019	0.600	0.355–1.013	0.056
Masaoka-Koga staging						
I + II	Reference			Reference		
III + IV	0.547	0.345–0.866	0.010	0.583	0.366–0.929	0.023
Surgical approach						
OT	Reference					
VATS	1.271	0.235	0.856–1.886			
Resection status						
R0	Reference					
None R0	0.493	0.198–1.224	0.127			
Perioperative complications	0.912	0.548–1.518	0.723			
Postoperative therapy	0.872	0.589–1.2	0.495			

Variables	Univariate model			Multivariate model		
	OR	95% CI	*P* value	OR	95% CI	*P* value
Sex						
Female	Reference					
Male	0.972	0.666–1.420	0.884			
Age	1.006	0.993–1.020	0.357			
Preoperative course						
< 12 month	Reference					
≥ 12 month	0.823	0.498–1.359	0.447			
Application of hormone	0.821	0.414–1.628	0.572			
Application of immunosuppressants	1.001	0.501–1.997	0.998			
Osserman classification						
I + IIA	Reference					
IIB + III + IV	0.836	0.569–1.2	0.361			
WHO pathological classification						
A + AB + MNT	Reference			Reference		
B + MIX	0.508	0.321–0.904	0.019	0.575	0.341–0.970	0.038
TNM staging						
I + II	Reference			Reference		
III + IV	0.612	0.380–0.988	0.045	0.649	0.401–1.053	0.080
Surgical approach						
OT	Reference					
VATS	1.271	0.856–1.886	0.235			
Resection status						
R0	Reference					
None R0	0.493	0.198–1.224	0.127			
Perioperative complications	0.912	0.548–1.518	0.723			
Postoperative therapy	0.872	0.589–1.2	0.495			

The Cox regression analysis of the proportion of complete stable remission is shown below. Univariate COX regression analysis indicated that older age (*P* = 0.044), preoperative course longer than 12 months (*P* = 0.092), Osserman staging IIA + IIB + III + IV (*P* = 0.063) and Video-assisted thoracoscopic surgery (VATS) (*P* = 0.031) were risk factors for achieving CSR. The result of multivariable COX regression analysis showed that thymoma patients with MG with Osserman staging IIA + IIB + III + IV (*P* = 0.011) were not prone to achieving CSR (Table 5).

**Table 5:** Analysis of influencing factors for achieving CSR in thymoma patients with MG

Variables	Univariate model			Multivariate model		
	OR	95% CI	*P* value	OR	95% CI	*P* value
Sex						
Female	Reference					
Male	1.006	0.541–1.870	0.985			
Age	0.977	0.954–1.000	0.051	0.978	0.953–1.004	0.092
Preoperative course						
< 12 month	Reference					
≥ 12 month	0.350	0.108–1.137	0.081	0.413	0.126–1.352	0.144
Application of hormone	0.863	0.266–2.799	0.806			
Application of immunosuppressants	0.272	0.037–1.984	0.199			
Osserman classification						
I	Reference					
IIA + IIB + III + IV	0.526	0.267–1.036	0.063	0.481	0.242–0.956	0.037
WHO pathological classification						
A + AB + MNT	Reference					
B + MIX	1.492	0.531–4.194	0.448			
Masaoka-Koga staging						
I + II	Reference					
III + IV	0.746	0.355–1.566	0.438			
TNM staging						
I + II	Reference					
III + IV	0.683	0.302–1.545	0.360			
Surgical approach						
OT	Reference					
VATS	0.534	0.2877–0.995	0.048	0.610	0.321–1.157	0.130
Resection status						
R0	Reference					
None R0	0.785	0.189–3.256	0.739			
Perioperative complications	1.010	0.445–2.289	0.982			
Postoperative therapy	1.232	0.626–2.424	0.546			

These patients were divided into two groups based on whether they had myasthenia gravis. Compared between the two groups, there were no significant differences in sex, tumor size, whether thymic hyperplasia or atrophy, preoperative course, surgical approach, TNM staging, Masaoka-Koga staging, or postoperative therapy (*P* > 0.05). Thymoma patients with myasthenia gravis were younger (*P* = 0.003), had longer operating duration (*P* = 0.003), and were more common in the WHO classification B-type (*P* = 0.000). Furthermore, they are more prone to perioperative complications (*P* = 0.032).

## Discussion

The incidence of thymic epithelial tumors is extremely low, typically 1.3 to 3.9 per 1, 000, 000 [7–10]. It is very rare in children and the incidence gradually increases with age, reaching a peak in the seventh decade of life [9]. We followed 242 patients with thymic epithelial tumors in Beijing Hospital from November 2011 to May 2019, recorded their long-term prognosis condition, and analyzed the factors affecting the prognosis of surgery.

Survival from malignant epithelial tumors of thymus diagnosed in Europe during the period 2000–2007 was 84% at 1 year and 64% at 5 years [10]. The 5-year survival rate is not high,probably due to the fact that their subset of patients included some who did not undergo surgery (not described in detail in the article). The 10-year overall survival (OS rate) for stage WHO type A, AB, and B1 was 90–95% [11, 12].The 5-year survival rates for stage WHO type B2, B3, and C were respectively 75%, 70%, and 48%. For patients with complete tumor resection, the 5-year survival rates for stage I, II, III, and IV [13] were 90%, 90%, 60%, and 25%, respectively. In our study, the 5-year survival rates of type A,AB,B1,B2,B3 and C patients were 100%, 95.5%, 92.6%, 88.1%, 81.8% and 75.0%.The 5-year survival rates of stage I, IIA, IIB, III and IVA patients were 95.3%,97.4%, 97.3%,80.8% and 50.9%, respectively. The overall 5-year survival rate was 91.1%.

Most studies showed Masaoka-Koga staging, TNM staging and resection status of tumors were independent risk factors for prognosis. However, in our study, multivariate COX regression analysis suggested that only thymoma recurrence was the independent risk factor for survival. In our center, thymoma patients with MG were carefully treated in the specialized MG wards of the Department of Neurology preoperatively and postoperatively. Moreover, they were followed up more closely after discharge and were admitted to the hospital whenever problems arose. Therefore, MG in our study did not affect postoperative prognosis. Several studies showed MG was a poor prognostic factor [14]. Many studies, however, have shown that MG symptoms can lead to the early detection of thymic epithelial tumors. MG may be a protective factor for thymic epithelial tumors, allowing for earlier detection and resection of tumors and a better prognosis [13, 15–19].

It is well accepted that the later MK stage and TNM stage, the more likely the tumor is to recur [20, 21]. Our study also found that the late stage of tumor is an independent risk factor for thymoma recurrence and younger patients were prone to recurrence after surgery, as some studies reported [20, 22, 23]. Studies reported that complete resection was an independent risk factor for thymoma recurrence [24, 25], but we didn't come to this conclusion. Patients with thymoma were recommended to undergo surgical resection even if R0 resection couldn’t be achieved. But it takes a long time for recurrence, which is significantly beneficial for the patient. Moreover, re-excision is suggested for recurrent thymomas to achieve long-term survival [3, 26–28]. Most of our patients who recurred also underwent secondary surgical resection, with a 5-year survival rate of 66%. The expected survival period after surgery for thymic epithelial tumors is long. Some experts suggested that the follow-up period should be more than 10 years [29]. There are even studies which demonstrate that OS should not be used as a prognostic evaluation indicator for thymic epithelial tumors because more than 50% of patients do not eventually die of the tumor itself [21].

MG is the most common complication of thymic epithelial tumors. Approximately 15–65% of TETs are complicated with MG [13, 16, 24, 30–36]. In contrast, approximately 10–15% of MG patients have a thymic epithelial tumor [30, 37]. The percentage of patients with MG in our group was relatively high (61.98%). Probably because we have a specialized ward for patients with myasthenia gravis in our hospital, all patients with MG routinely undergo chest CT and therefore more thymic epithelial tumors are detected. In our study, thymoma patients with MG were younger than patients without MG, suggesting that younger people with thymoma were more likely to develop MG, which was similar to the findings of many other studies [13, 16, 24, 35, 38, 39]. The operation duration was longer in patients with MG. Extended thymectomy was performed in thymoma patients with MG, which may take more time to remove the fatty tissue from the anterior mediastinum and caridiodiaphragmatic angle. Patients with MG may have poor expectoration, be prone to aspiration, and even develop myasthenic crisis following surgery. Therefore, the incidence of perioperative complications was higher in patients with MG, as seen in our study.

Different types of thymomas develop MG based on different mechanisms. [40]. Thymomas derived from different cells differ in the expression of AChR and some proteins that share epitopes with TAMG-related autoantibody targets. Additionally, different types of thymomas exhibit different MG-related up-and down-regulation pathways [41]. According to the WHO pathological classification, type B is more likely to be combined with MG [42–45]. The mechanism of thymoma complicating MG is not yet clear nowadays. The expression of the NM-F protein in type B thymoma in conjunction with MG can improve T cell responses but not in types A and AB, which may be an effective factor for type B being more likely to be complicated with MG [29].

In our study, type B and late stage of tumors were independent predictors of poor outcome after thymectomy in thymoma-associated MG patients. Thymoma patients with advanced stages were more prone to recurrence. After the recurrence of thymoma, the symptoms of MG will probably worsen or recur after improvement, thus affecting the therapeutic effect of MG. In addition, thymoma with advanced stage and more malignant biological behavior is not easily removed completely by surgery, which will result in a small amount of residual thymoma tissue or cells in the patient's body that can still secrete acetylcholine receptor antibodies, which is not conducive to the improvement of MG symptoms.

Based on the documented statistics, the rate of complete stable remission of myasthenia gravis is about 16–59.5% postoperatively [46–48]. In our study, the postoperative CSR rate was 30.5% in MG patients with thymoma at follow-up. Mutivariate COX regression analysis indicated that patients with Osserman typing IIA + IIB + III + IV would be less likely to achieve CSR status postoperatively. With longer follow-up, the CSR of MG patients gradually increased. Patients with advanced Osserman stage suffered more severe MG symptoms and, subsequently, postoperative improvement decreased.

CCI was proposed in 1987 by Charlson in the United Kingdom to assess the impact of co-morbidities and primary underlying disease under treatment on patients’ future survival [49]. It is a proven, simple, and readily applicable method to estimate the risk of death from comorbid disease and has been widely used as a predictor of long-term prognosis and survival [50]. Many studies [51, 52] have analyzed the relationship between CCI and the incidence of postoperative mortality, and found a significant correlation. There is no report on the correlation between CCI and the prognosis of patients with thymic epithelial tumors. In this study, we evaluated the preoperative CCI of each patient. After the multivariate analysis, it did not become an independent risk factor for survival or recurrence, although *P* value was 0.016 in the univariate analysis (Table 2). Our follow-up time is 3–11 years, with an average of 70.5 months. The time is relatively close to that of other studies. In our study, CCI did not become an independent risk factor for long-term survival of patients. The possible reasons are as follows: (1) There were not much deaths in our study (23 cases), fewer patients died from complications, most of whom died because of recurrent thymic epithelial tumors or MG-related problems. After all, MG was not included in CCI statistics in this study; (2) Thymic epithelial tumors are malignant tumors, and the recurrence leads to a significant reduction in the survival of patients, which may make the impact of CCI on the long-term survival of these patients undetectable in our study; (3) Both the sample size and follow-up time were insufficient, which may not reflect the effect of CCI on long-term prognosis. In conclusion, the relationship between CCI and thymoma epithelial patients needs further study.

Several limitations should be pointed out in this study. First, the patients' procedures were not performed by the same surgeon, and some variation in the operator's surgical technique may have led to errors in the results. Second, this was a retrospective study, and there were many influencing factors inevitably which may have influenced the results of this study. Third, all patients in this study were from one single center. The characteristics of patients from different centers were also different. Therefore, the patient-selection bias was unavoidable. Fourth, the follow-up period was relatively short in our study. It is difficult to evaluate sufficiently the prognosis of patients with thymoma.

## Conclusion

Surgical treatment of thymoma provides a favorable long-term prognosis. Younger age and advanced stage were independent risk factors for RFS of patients with TETs. And Charlson Comorbidity Index and recurrence of thymoma were independent risk factors for OS. For thymoma patients with MG, WHO classification type B and advanced stage were independent predictors of poor outcomes of MG treatment after thymectomy.

## Supplementary Information

**Additional file 1: Figure S1.** The K-M survival curves of OS and RFS for patients.

## Data Availability

The datasets used during this study are available from the corresponding author upon reasonable request.

## Abbreviations

TETs/TET: Thymic epithelial tumors
MG: Myasthenia gravis
OS: Overall survival
RFS: Relapse-free survival
CSR: Complete stable remission
PR: Pharmacologic remission
MM: Minimal manifestations
I: Improved
U: Unchanged
W: Worse
E: Exacerbation
D: Dead
VATS: Video-assisted thoracoscopic surgery
OT: Open thoracotomy
