# Supplementary figures and images for: Postoperative clinical outcomes of patients with thymic epithelial tumors after over-3-year follow-up at a single-center
Source: J Cardiothorac Surg. 2023 Feb 21;18:77. doi: 10.1186/s13019-023-02169-6 (PMC9942311; doi:10.1186/s13019-023-02169-6)

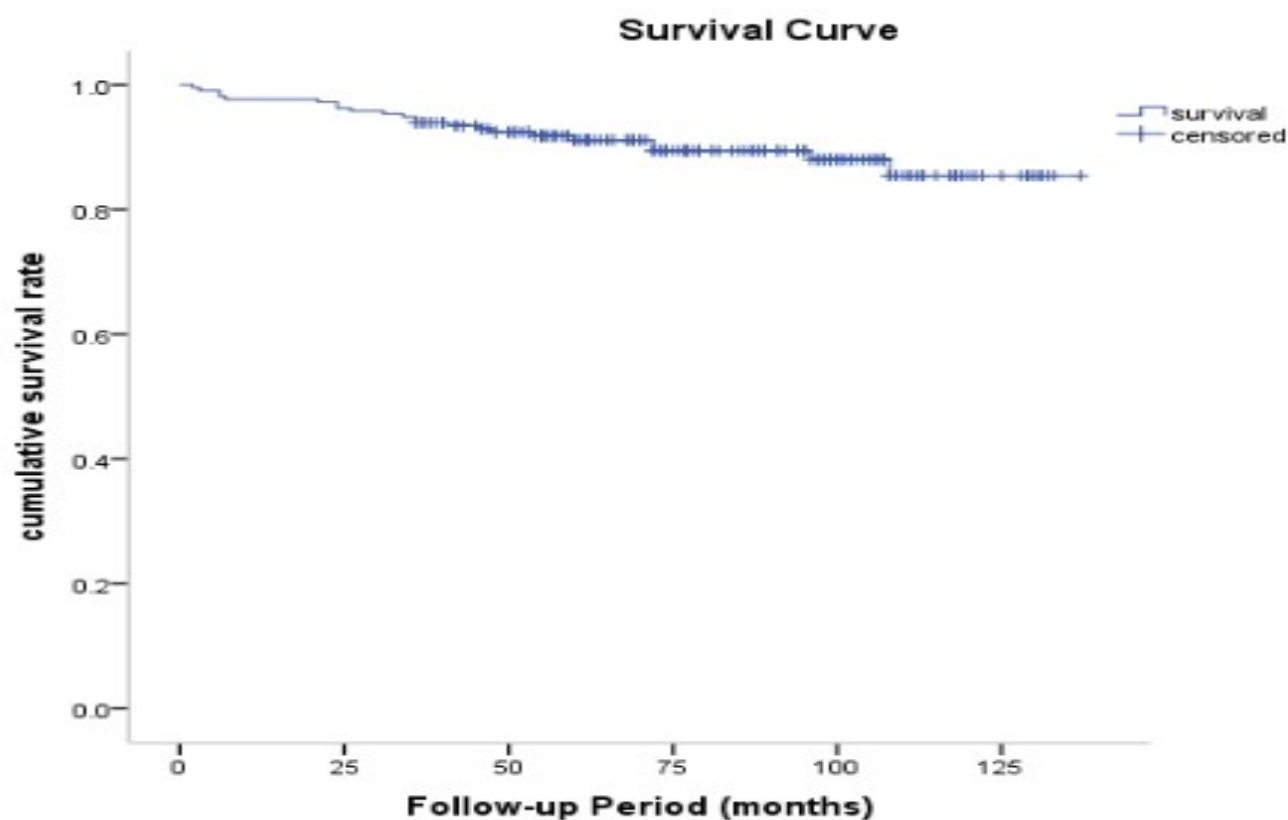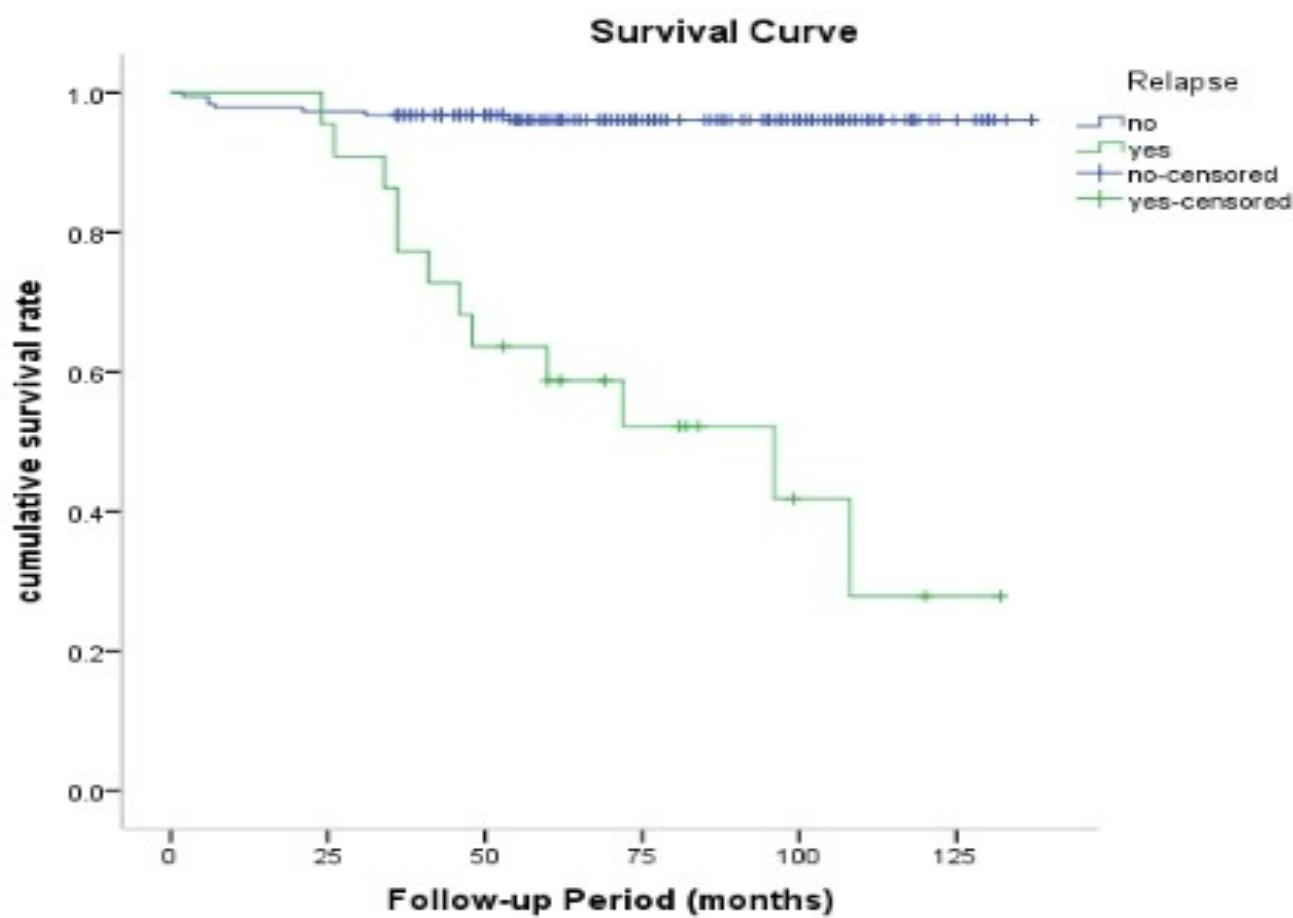

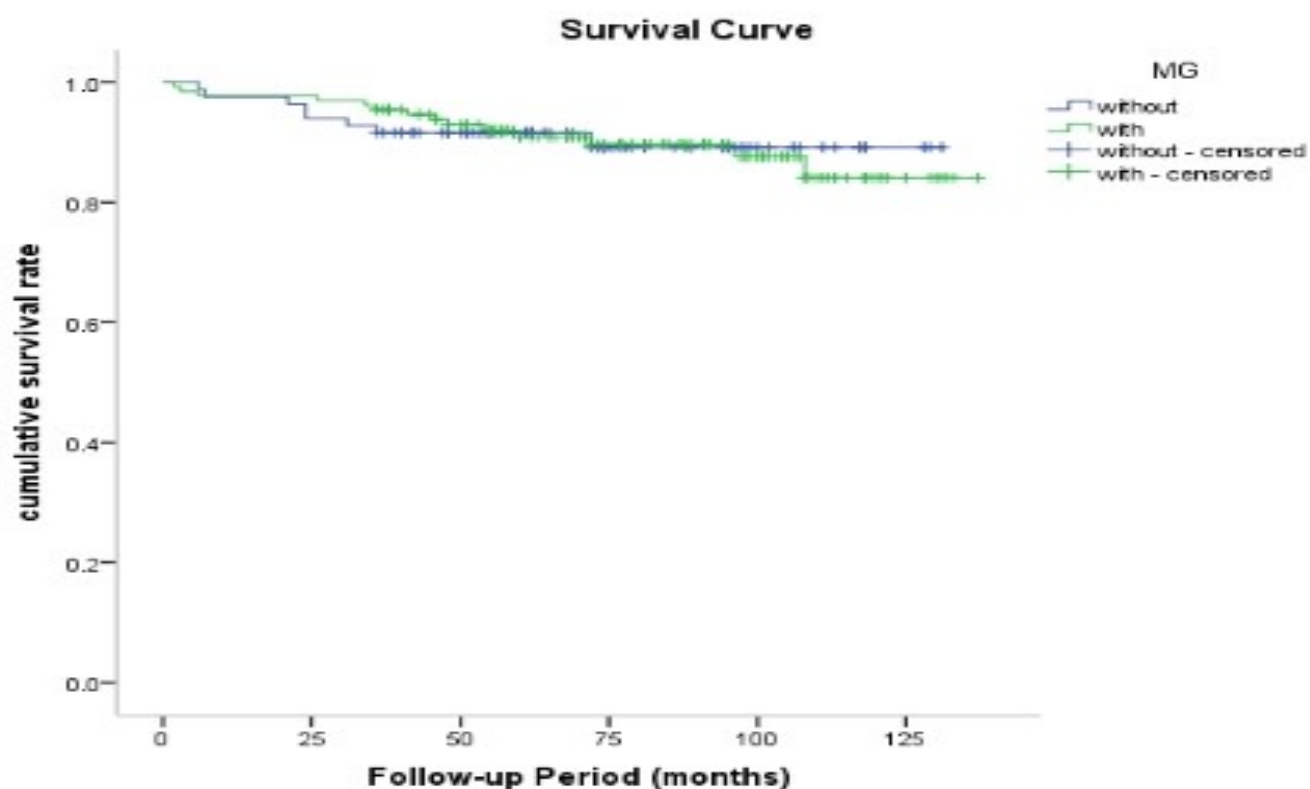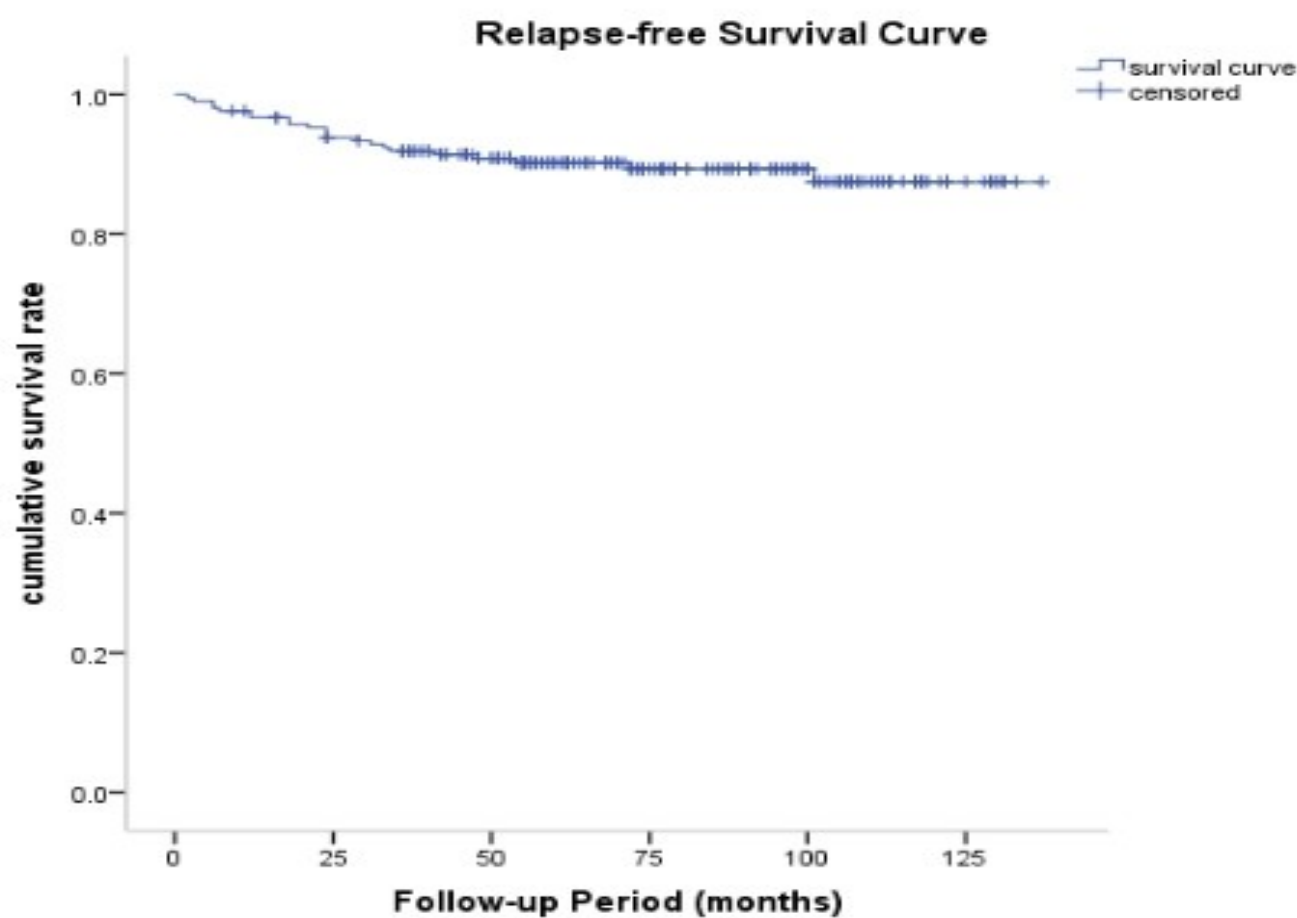

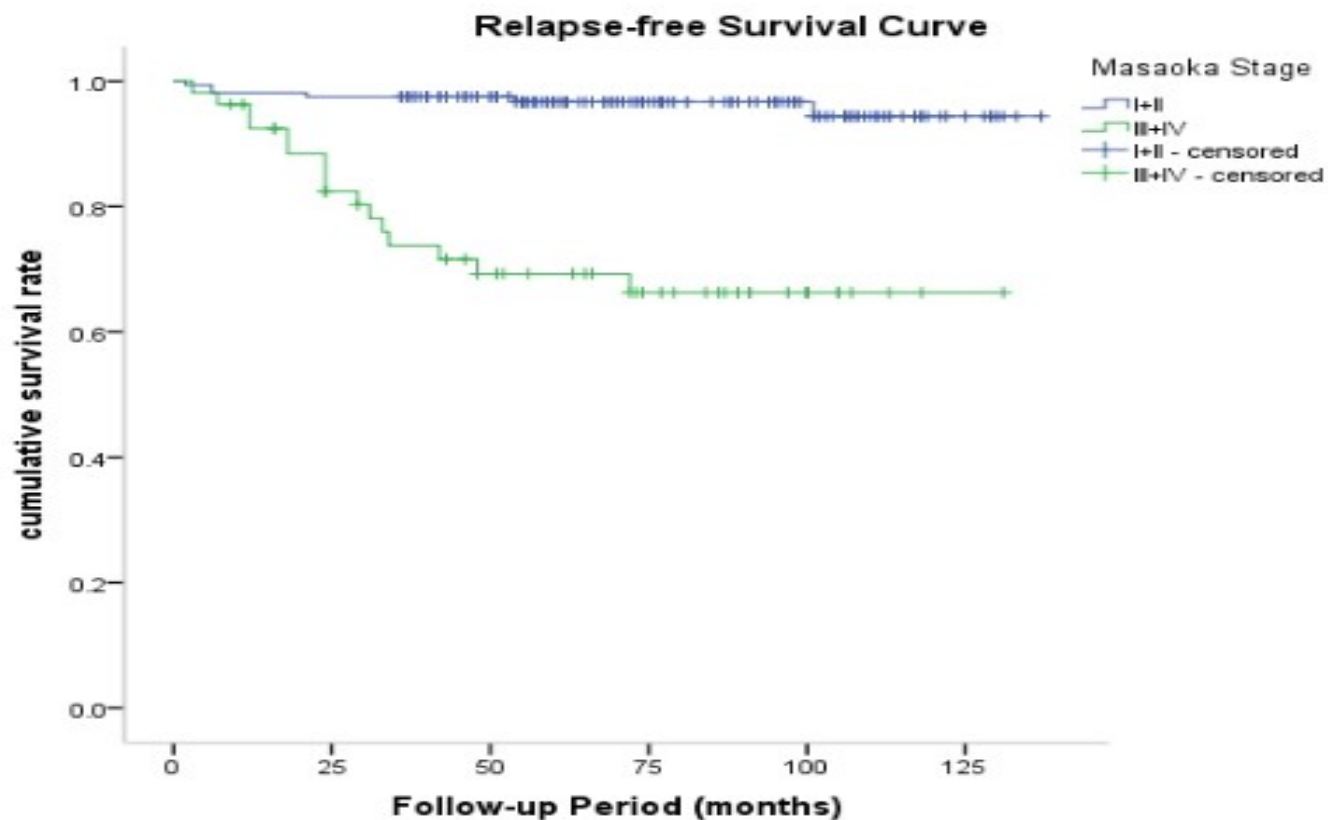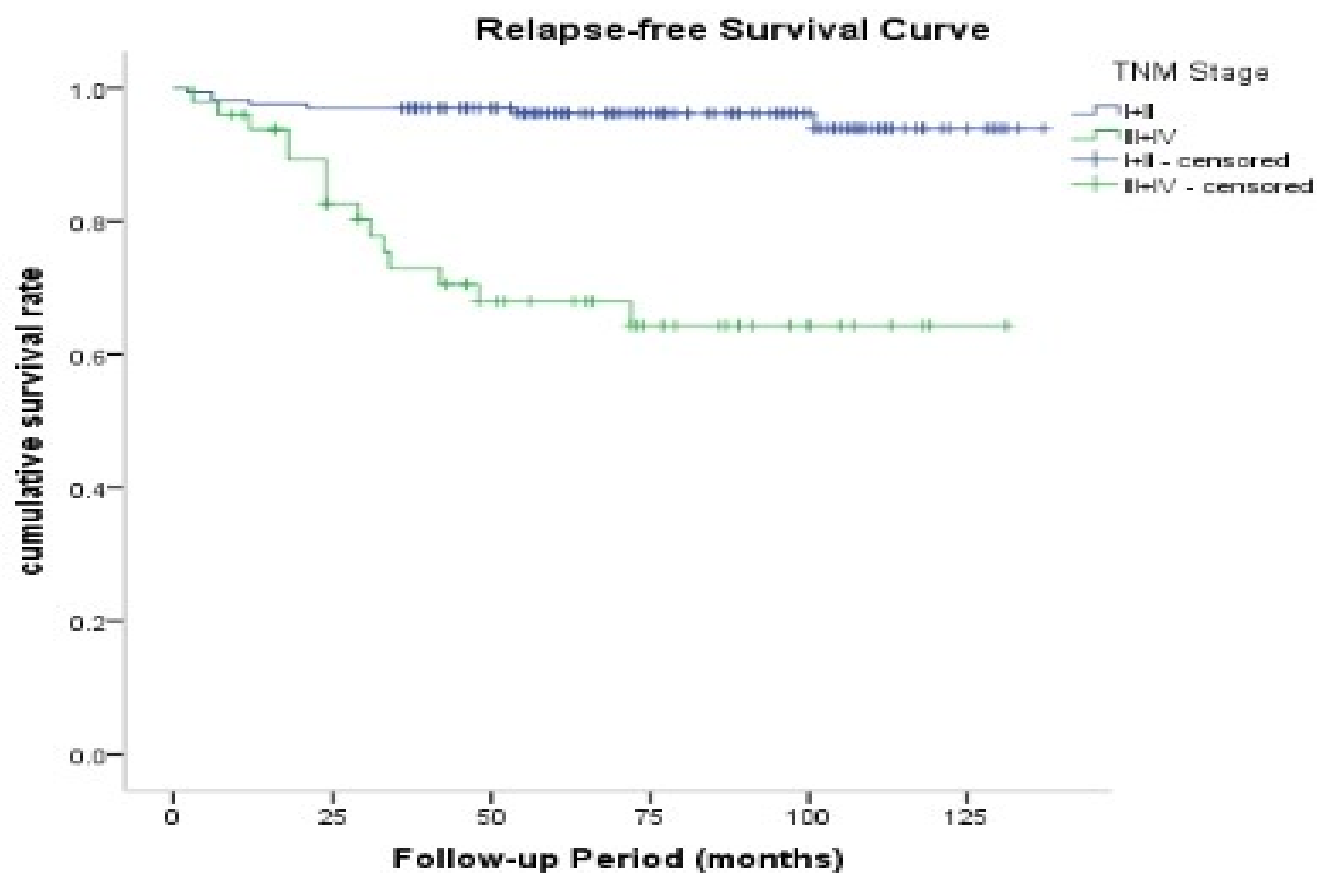

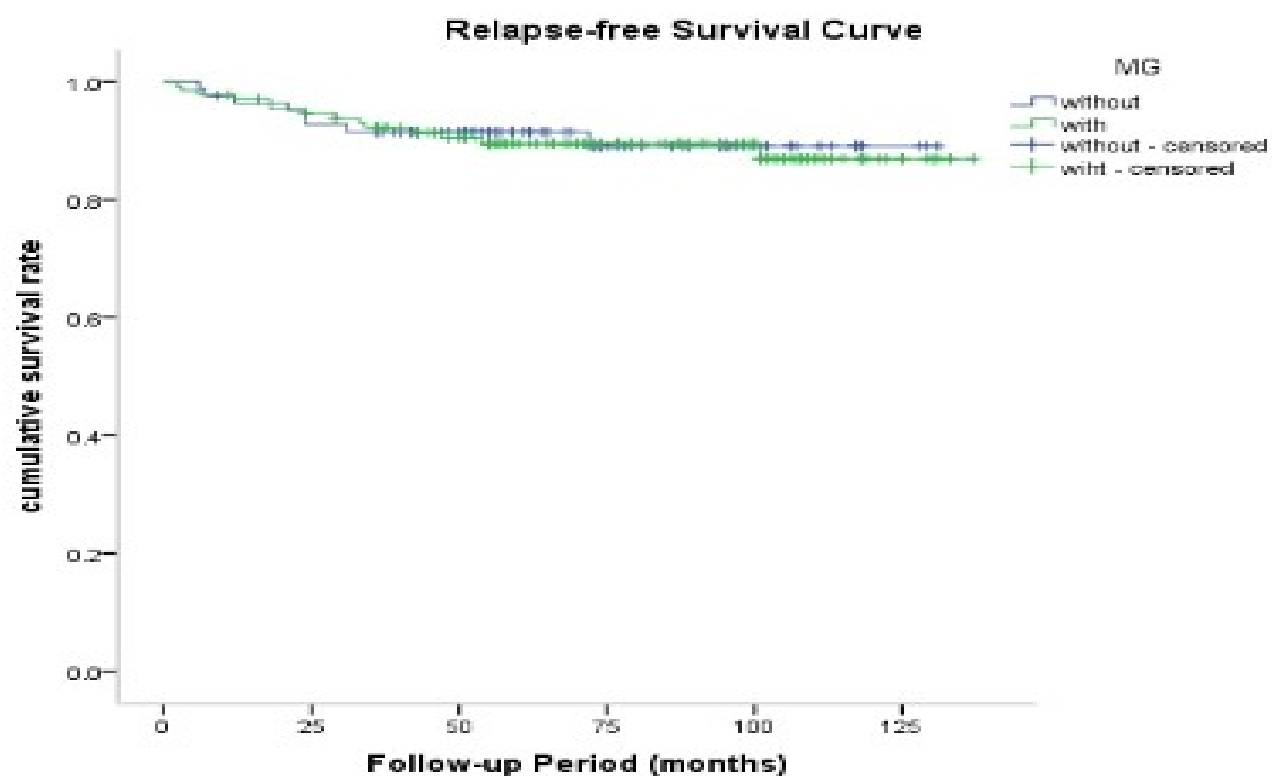

Supplement: Supplementary file 1 — Additional file 1: Figure S1. The K-M survival curves of OS and RFS for patients. [file 13019_2023_2169_MOESM1_ESM.pdf]
